# TRP Channels in Oxidative Stress Signalling

**DOI:** 10.3390/cells12091251

**Published:** 2023-04-26

**Authors:** Lin-Hua Jiang, Xiaoqiang Yao, Bilal Çiğ

**Affiliations:** 1 Sino-UK Joint Laboratory of Brain Function and Injury of Henan Province, Department of Physiology and Pathophysiology, School of Basic Medical Sciences, Xinxiang Medical University, Xinxiang 453003, China; 2 School of Biomedical Sciences, Faculty of Biological Sciences, University of Leeds, Leeds LS2 9JT, UK; 3 School of Biomedical Sciences, The Chinese University of Hong Kong, Hong Kong, China; 4 Department of Physiology, Medicine Faculty, Kirsehir Ahi Evran University, Kirsehir 40100, Turkey

## 1. Introduction

It is well established that the accumulation of high levels of reactive oxygen species (ROS), due to excessive generation of ROS and/or impaired antioxidant capacity of cells, can result in oxidative stress and cause oxidative damage to cells and their functions. While oxidative damage has been long and well documented as a conspicuous and common feature in numerous pathological conditions, compelling evidence exists demonstrating that oxidative stress is an important factor in the pathogenesis of many of these conditions. Mammalian cells express a large family of transient receptor potential (TRP) channels, which can be divided, based on sequence relatedness, into TRPA (ankyrin), TRPC (canonical), TRPM (melastatin), TRPML (mucolipin), TRPP (polycystin) and TRPV (vanilloid) subfamilies [1]. TRP channels display wide tissue and cell distributions and activation by multiple modalities. Several TRP channels, including TRPA1, TRPC6, TRPM2, TRPM7 and TRPV1, exhibit a salient sensitivity to oxidative stress [2]. Emerging evidence suggests that these TRP channels act as cellular redox sensors and play a role in transducing various oxidative stress-induced factors to the pathogenesis and progression of associated conditions, such as ischemic stroke, atherosclerosis, metabolic syndrome, diabetes and cancers. This Special Issue (SI) has collected seven papers that examined the role of TRP channels in multiple oxidative stress-related diseases.

## 2. Ischemic Stroke Brain Damage

Stroke is a common cause of mortality and morbidity. Ischemic stroke is the predominant type, causing neuronal death and brain tissue damage that are ultimately responsible for the loss of neurological functions. TRPM7 channel expression in neurons was previously reported to be upregulated by ischaemia-induced oxidative stress and the TRPM7 channel plays an important role in mediating neuronal death, tissue damage and neurological deficits. Zhang, P. et al. compared the role of the TRPM7 channel in parvalbumin (PV)-positive gamma-aminobutyric acid (GABA)ergic neurons and Ca^2+^/calmodulin-kinase II (CaMKII)-positive glutamatergic neurons in mediating brain damage following ischaemia/reperfusion (I/R) [3]. In wild-type (WT) mice, exposure to I/R led to a higher level of neuronal death, as well as a larger increase in TRPM7 expression in GABAergic neurons than in glutamatergic neurons in the penumbra of the mouse cortex. The survival rate after I/R was significantly improved and the infarction was reduced by conditional knockout of TRPM7 expression in GABAergic neurons (PV-TRPM7-KO) or in glutamatergic neurons (CaMKII-TRPM7-KO), with a higher survival rate and lower infarct volume in the PV-TRPM7-KO mice. The neuronal death of both GABAergic and glutamatergic neurons was attenuated by PV-TRPM7-KO, but only glutamatergic neuron death was mitigated by CaMKII-TRPM7-KO. In addition, I/R-induced neurological and motor deficits were significantly reduced by PV-TRPM7-KO, but not by CaMKII-TRPM7-KO. Furthermore, activation of astrocytes and microglia, indicated by increased expression of glial fibrillary acidic protein (GFAP) and ionized calcium binding adaptor molecule 1 (IBA1) and the production of tumour necrosis factor (TNF)-α, was attenuated in the PV-TRPM7-KO mice to a greater degree than that in the CaMKII-TRPM7-KO mice. Finally, activation of Akt, known to facilitate the recovery from I/R, was only observed in the PV-TRPM7-KO mice. The post-I/R levels of p53 expression, a key regulator of apoptosis that is inhibited by Akt, and cleaved caspase-3, a key factor driving apoptotic cell death, were reduced in both transgenic mice, with the reduction in the PV-TRPM7-KO mice being more noticeable in comparison with that in the CaMKII-TRPM7-KO mice. Taken together, these observations provide new insights into the role of the TRPM7 channel in mediating ischemic stroke brain damage.

Zong et al. presented a systemic review on TRPM2, another TRP channel that is activated in response to oxidative stress, in the pathogenesis of ischemic stroke [4]. They started with discussing the role of the TRPM2 channel in diverse conditions that are well established risk factors for ischemic stroke, including atrial fibrillation, hypertension, atherosclerosis, diabetes and thrombosis, before elaborating the cellular and molecular mechanisms by which the TRPM2 channel contributes to ischemic stroke brain damage. In the end, the authors briefly highlighted the potential of targeting the TRPM2 channel to develop medications mitigating ischemic stroke brain damage.

## 3. Peripheral Artery Disease

Peripheral artery disease (PAD) occurs when the lower extremity blood flow is impaired, due to progressive occlusion of large conductance arteries induced, e.g., by atherosclerosis, and retarded revascularization is a major cause of poor prognoses for PAD. Following their previous study showing that deletion of the TRPC6 expression facilitated vascular smooth muscle cell (VSMC) differentiation and arteriogenesis after hind limb ischaemia (HLI), a model of PAD [5], Shimauchi et al. identified 1-benzilpiperadine (1-BP) as an inhibitor of the TRPC6 (and TRPC3) channels, and examined the effects of treatment with 1-BP on HLI-induced changes in mice, as well as on VSMC differentiation [6]. Treatment of mouse aortic SMCs with 1-BP promoted VSMC differentiation. Treatment with 1-BP improved peripheral circulation, stimulated arterialization, and increased skeletal muscle mass and motor function after HLI in the WT mice, but not in the TRPC6-KO mice, suggesting that I-BP induced these beneficial effects by targeting the TRPC6 channel. Interestingly, treatment of hypercholesterolemic mice with endothelial dysfunction with 1-BP also facilitated blood flow recovery after HLI, indicating the retrograde interaction from VSMCs to the endothelium. This study demonstrated the potential of inhibiting the TRPC6 channel as a therapeutical strategy for treating PAD.

## 4. Atherosclerosis

Atherosclerosis is a chronic metabolic and inflammatory disease characterized by the formation of atherosclerotic plaques on the walls of medium- and large-sized arteries, leading to progressive narrowing of the arterial lumen, and represents a major cause of many cardiovascular disorders and ischemic stroke. Hypercholesterolaemia and excessive oxidative stress in arterial walls are among the main factors promoting atherosclerosis. Zhang, Y. et al. examined the role of the TRPM2 channel in atherosclerosis, mainly using the hypercholesterolaemia-induced mouse model consisting of mice fed with a high cholesterol diet and overexpressing PCSK9, which promotes platelet activation and leukocyte recruitment [7]. They first demonstrated that TRPM2 functions as a ROS-activated Ca^2+^-permeable channel on the cell surface of endothelial cells, macrophages and SMCs, the major three types of cells that participate in the early and later stages of atherosclerosis. In the atherosclerotic plaques from hypercholesterolaemia-induced mice and in atherosclerotic carotid arteries and intact arteries of patients, TRPM2 expression was significantly elevated. In mice, atherosclerotic plaque formation in both whole aortas and aortic-root thin sections, and the expression of CD68, α-SMA and PCNA in the plaque regions were reduced by TRPM2-KO, demonstrating a significant role for the TRPM2 channel in stimulating macrophage infiltration and SMC migration into the lesion area. In addition, the levels of ICAM-1, MCP-1 and TNF-α and the ROS production in the plaque regions were also decreased by TRPM2-KO, suggesting a role of the TRPM2 channel in promoting monocyte adhesion and inducing vascular inflammation. The production of inflammatory cytokines in bone marrow-derived macrophages and primary arterial endothelial cells, and the ROS production in endothelial cells, macrophages and SMCs were also suppressed by TRPM2-KO. Consistently, the atherosclerotic legions, the levels of CD68, α-SMA and ICAM-1 in the aorta, and the production of proinflammatory cytokines and ROS by macrophages and endothelial cells in ApoE-deficient mice, another model of atherosclerosis, were mitigated by treatment with ACA, a TRPM2 inhibitor. Collectively, these results indicate that the TRPM2 channel is an important mechanism transducing ROS production induced by atherosclerosis-promoting pathological factors to contribute to inflammation and atherosclerosis.

## 5. Metabolic Syndrome

Metabolic syndrome is a complex pathological condition characterized by visceral adiposity, insulin resistance, arterial hypertension and dyslipidaemia, with hypoadiponectinaemia, inflammation and oxidative stress known as important pathological factors. Araújo et al. examined the literature about the role of the TRP–oxidative stress axis in the pathogenesis and progression of metabolic syndrome, focusing on TRPV1, TRPA1 and TRPC5 in regulating the function of metabolic tissues and cells, and their connections with the central nervous system. They also discussed the potential of such TRP channels as therapeutical targets for metabolic syndrome [8].

## 6. Diabetic Cataract

Cataracts are a serious complication of diabetes that can severely impair vision. Long-term hyperglycaemia due to diabetes results in elevated ROS production and oxidative stress, and loss of lens epithelial cells plays a key part in inducing cataracts. Chen et al. investigated the expression of the TRPV2 channel in lens epithelial cells and its role in mediating the development of diabetic cataracts, particularly high glucose-induced apoptosis of lens epithelial cells [9]. The TRPV2 expression was demonstrated by immunofluorescent imaging and Western blotting, and its expression level was significantly higher in lens epithelial cells of patients with diabetic cataracts than that in senile cataracts, and in human lens epithelial cells and primary rat lens epithelial cells cultured in the presence of high glucose concentrations. The rise in intracellular Ca^2+^ levels evoked by 2-APB, a TRPV2 channel agonist, was also significantly higher in these cells, and was blocked by prior treatment with ruthenium red, a TRPV2 inhibitor, or small interfering (si)RNA for TRPV2 (TRPV2-siRNA). Prolonged culturing of human and rat lens epithelial cells in high glucose media resulted in increased apoptosis, which was inhibited by treatment with TRPV2-siRNA. High glucose-induced upregulation of the TRPV2 expression, increase in 2-APB-induced Ca^2+^ responses and apoptosis in lens epithelial cells were suppressed by treatment with Tempol, a ROS inhibitor, consistent with the importance of ROS in mediating high glucose-induced effects. These results suggest that the TRPV2 channel is an important mechanism that transduces high glucose-induced oxidative stress to induction of apoptosis of lens epithelial cells due to diabetes.

## 7. Cutaneous Melanoma

Cutaneous melanoma is the most common and aggressive subtype of melanoma, with oxidative stress known to associate with melanocyte transformation and tumour-associated macrophages critically implicated in the pathogenesis. The TRPA1 expression was reported in melanoma cell lines. De Logu et al. assessed the infiltration and state of macrophages, 4-HNE, a final end-product of oxidative stress, and the TRPA1 expression in tissue samples of human common dermal melanocytic nevi, dysplastic nevi, and thin and thick cutaneous melanomas [10]. The number of CD163-positive M2-type macrophages and the 4-HNE level in the intratumoural and peritumoural regions progressively increased with tumour severity, but the TRPA1 expression was not altered. In SK-MEL-28 and WM266-4 melanoma cells, application of allyl isothiocyanate, a TRPA1 agonist, or H_2_O_2_ evoked concentration-dependent increases in intracellular Ca^2+^ levels that were inhibited by prior treatment with A967079, a TRPA1 channel-specific antagonist. In addition, application of allyl isothiocyanate promoted the production of H_2_O_2_ in both types of melanoma cells, which was prevented by treatment with A967079 or TRPA1-siRNA. Thus, this study revealed the activation of the TRPA1 channel in melanoma cells by ROS released from macrophages as a mechanism amplifying oxidative stress signalling.

In summary, the original studies and reviews collected in this SI provide new insights into the important role of oxidative stress-sensitive TRP channels as signal transduction mechanisms that couple diverse oxidative stress-induced pathological factors to the pathogenesis of associated diseases.
